# Antibody diversity in IVIG: Therapeutic opportunities for novel immunotherapeutic drugs

**DOI:** 10.3389/fimmu.2023.1166821

**Published:** 2023-03-28

**Authors:** Stephan von Gunten, Christoph Schneider, Lejla Imamovic, Guy Gorochov

**Affiliations:** ^1^ Institute of Pharmacology, University of Bern, Bern, Switzerland; ^2^ Sorbonne Université, Inserm, Assistance Publique Hôpitaux de Paris (AP-HP), Pitié-Salpêtrière Hospital, Paris, France

**Keywords:** antibodies, intravenous immune globulin (IVIg), subcutaneous immunoglobulin, IgA, immunotherapy

## Abstract

Significant progress has been made in the elucidation of human antibody repertoires. Furthermore, non-canonical functions of antibodies have been identified that reach beyond classical functions linked to protection from pathogens. Polyclonal immunoglobulin preparations such as IVIG and SCIG represent the IgG repertoire of the donor population and will likely remain the cornerstone of antibody replacement therapy in immunodeficiencies. However, novel evidence suggests that pooled IgA might promote orthobiotic microbial colonization in gut dysbiosis linked to mucosal IgA immunodeficiency. Plasma-derived polyclonal IgG and IgA exhibit immunoregulatory effects by a diversity of different mechanisms, which have inspired the development of novel drugs. Here we highlight recent insights into IgG and IgA repertoires and discuss potential implications for polyclonal immunoglobulin therapy and inspired drugs.

## Introduction

Polyclonal immunoglobulin (Ig) preparations, administered *via* intravenous (IVIG) or subcutaneous (SCIG) routes, are successfully used as replacement therapy in immunodeficiencies and for the treatment of select autoimmune or inflammatory disorders (1). These preparations consist of pooled human IgG derived from the plasma of thousands of donors and in contrast to therapeutic monoclonal antibodies encompass an immense range of reactivities (2, 3). Pathogen-specific antibodies in IVIG protect immunodeficient patients from infection, and we observed that IVIG even contains antibodies to host attachment sites of viruses, bacteria, and bacterial toxins, which might prevent adhesion or limit sequelae of infectious disease (2). In the recent years, high-throughput methods and mechanistic studies have further expanded our insights into antibody repertoires in health and disease and have revealed a plethora of canonical and non-canonical effector mechanisms by which polyclonal preparations exert therapeutic effects in infectious or autoimmune diseases. Insights into IVIG mechanisms of action have inspired the development of novel therapeutic agents or of alternative or modified polyclonal preparations with broader or shifted reactivity profiles. In this review article, we provide an overview on these recent developments and discuss challenges and benefits of diversity in respect to therapy using polyclonal immunoglobulin preparations.

## Canonical and noncanonical functions of antibodies

Activities of antibodies are executed by both major regions of the immunoglobulin, the fragment antigen binding (Fab), consisting of the light chain and part of the heavy chain, and the constant fragment crystallizable (Fc) domain. The variable Fab fragment is responsible for specific molecular recognition and binding of the antigen (epitope-paratope interaction). Beyond targeting specific antigens, the Fab fragment is responsible for the execution of important effector functions, such as direct neutralization of toxins or pathogens, agglutination, or blocking of ligand-receptor interactions (4, 5). Fab-mediated functions rely on structural properties of the complementarity determining region, which depend on variable region gene usage, and somatic mutations acquired during the process of affinity maturation. Fc-mediated effector functions are determined by the immunoglobulin class (IgG, IgA, IgM, IgD and IgE) or subclass (in humans: IgG1-4; IgA1or IgA2), respectively, which also influence antibody interactions with immune proteins such as Fc receptors (FcR), TRIM21, or complement factors. Depending on class and context, Fc-mediated effects result in the activation or regulation of immune cells (e.g. phagocytosis, degranulation and secretion of inflammatory mediators and effector molecules), and of non-cellular immune responses (e.g. complement activation) (4, 6).

While these classical functions of antibodies are commonly known, a large body of research revealed a range of alternative antibody activities, which have also been referred to as ‘noncanonical’ functions (6). Such noncanonical capabilities of immunoglobulins include antibody-mediated catalysis and proteolytic activities (6, 7), direct pathogen inactivation in the absence of effector systems, modification of gene expression and metabolism, reactive oxygen species [ROS] vulnerability, bactericidal pore formation and disruption of membrane integrity, and cofactor activities (e.g. in pathogen neutralization) (reviewed in (6)). Furthermore, the human repertoire of IgG antibodies contains functional antibodies specific for immunoregulatory receptors such as Fas (8–10) or Siglecs (5, 11, 12), that exhibit the capacity to regulate the survival of immune cells (13). Many noncanonical functions of antibodies are exclusively exerted by the variable region of the antibody (6), and their occurrence may thus differ among individuals depending on their private antibody repertoire. However, some antibody activities that could be classified as noncanonical can also be mediated by the constant antibody region. For instance, regulatory T cell (Treg) epitopes, also known as Tregitopes, contained in the highly conserved framework regions of Fab and Fc domains, are thought to expand and activate regulatory T cells subsequent to their processing and presentation on human class II major histocompatibility complex (MHC) molecules by antigen-presenting cells (APCs) (14). Indeed, it has been postulated that Tregitopes in Fab and Fc segments contribute to the immunoregulatories activities of IVIG (14). However, canonical and noncanonical functions of antibodies may act in parallel to collectively exert manifold immunoactivatory and regulatory tasks needed to maintain tissue integrity and homeostasis in health or in pathologic body states (15–18), which eventually allows for - and even requires - a certain degree of ‘benign autoimmunity’ (19, 20).

## Diversity – the added value of polyclonal immunoglobulin preparations

Commercial IVIG and SCIG preparations consist of the pooled antibodies, typically isolated serum IgG, and thus reflect the polyclonal antibody repertoire of the donor population. Antibody replacement therapy for patients with immunodeficiencies relies on the wide repertoire of immunoglobulins in these preparations (2, 3). Polyclonal immunoglobin preparations may compensate for quantitative or qualitative repertoire defects in immunodeficient patients, such as the decrement of distinct anti-microbial and even tumor-specific antibodies (21). Indeed, in common variable immunodeficiency (CVID) patient sera, besides lack of antimicrobial antibodies, we observed low IgG reactivities to tumor-associated carbohydrates antigens (TACAs) expressed on CVID-associated malignancies (21), such as Thomson-Friedenreich antigen in gastric cancer (22, 23), or GM1 in non-Hodgkin lymphoma (24). While the risk of CVID patients to develop specific malignancies could generally be associated with recurrent infection-related inflammation, the evidence of reduced levels of tumor-directed antibodies in CVID patients (21), supports the notion of impaired tumor immune surveillance in primary antibody immunodeficiencies (PAD) (25). Taken together, these observations suggest that IVIG replacement therapy may not only protect from infectious disease but contribute to protection from malignancies *via* tumor-specific antibodies that restore aberrant immune surveillance in immunodeficient patients. Profiling of individual patient antibody repertoires may lead to more personalized treatments (21, 26), potentially with specific immunoglobulin preparations (27). For instance, patients with selective IgA deficiency (SIgAd) that lack IgA but can produce IgG antibodies, present with an exacerbated anti-autologous microbiota IgG response (28). As a result, serum IgG from a SIgAd patient interacts more broadly with its own microbiota than current IVIG preparations (28). Therefore, an IVIG formula better suited to CVID and other conditions at risk of bacterial translocation might ideally consist of pooled IgG from SIgAd patients rather than from healthy individuals. Such preparations might be more effective than current IVIG as they would be naturally enriched in IgG specificities directed at species prone to bacterial translocation from various body sites, including the respiratory tract. Gut dysbiosis and respiratory tract infections are among common morbidities affecting CVID and symptomatic SIgAd patients.

**Figure 1:**
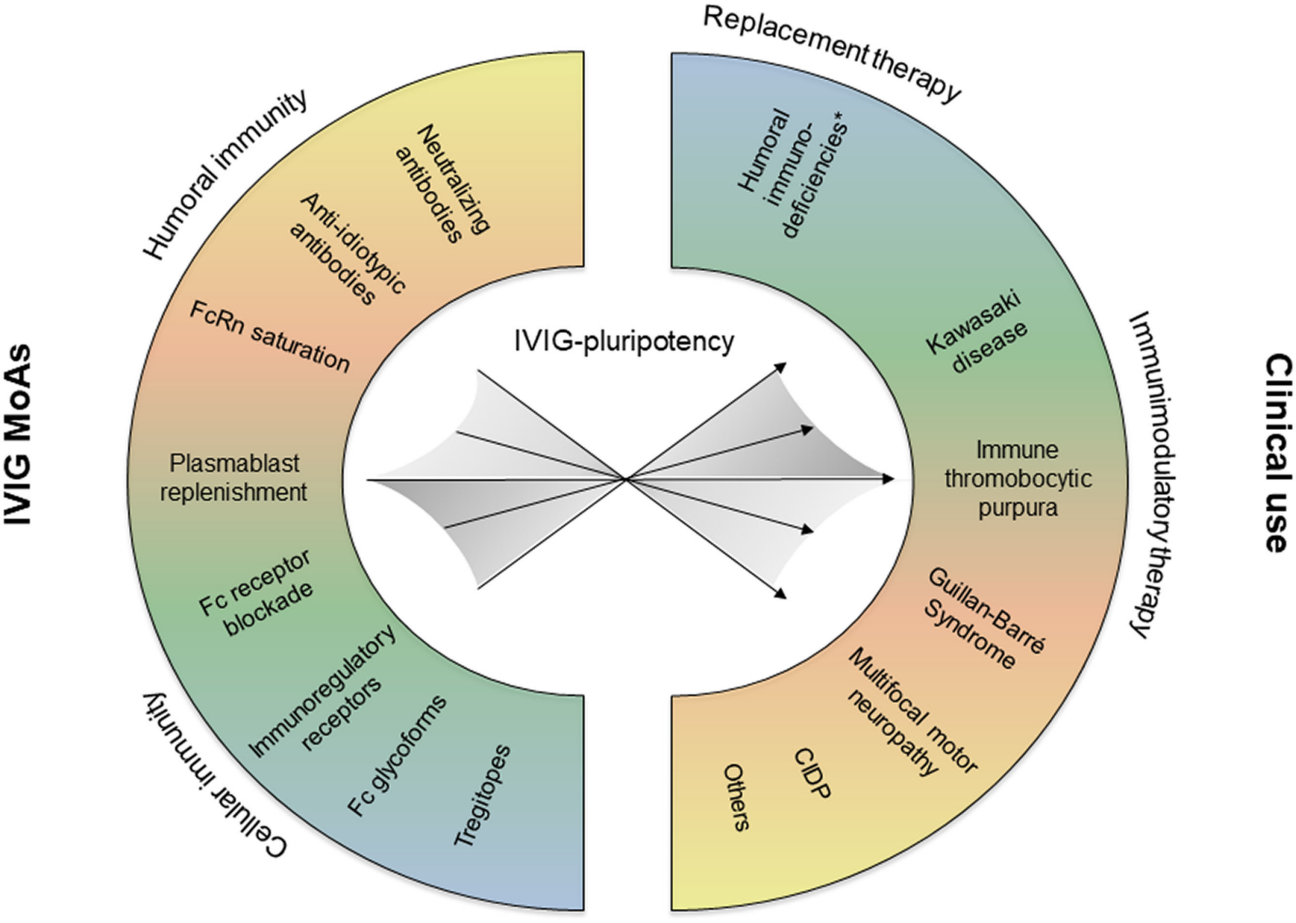
Depending on the pathophysiology of the treated disease, different IVIG mechanisms of action (MoAs) may be effective, and eventually act in concert. CIDP, chronic inflammatory demyelinating polyneuropathy.

As a high-dose therapy, IVIG and SCIG preparations are also used for the treatment of autoimmune and inflammatory disorders (1). Studies over the period of several decades revealed pleiotropic anti-inflammatory mechanisms of action (MoAs) of polyclonal immunoglobulin preparations (15, 29). The wide spectrum of reported MoAs includes Fc receptor blockade, neonatal Fc receptor (FcRn) saturation, induction of inhibitory FcgRIIB receptors, induction of B cell hyporesponsiveness, effects of specific Fc glycoforms (sialylation), Tregitopes, idiotypic-anti-idiotypic network restoration, neutralizing antibodies (e.g. microbial toxins, complement factors, cytokines), and antibodies exerting immunoregulatory effects on distinct leukocyte subsets (5, 13, 14, 30–33). Depending on the pathophysiology of a select disease, different MoAs may act in concert (15, 34), yet distinct mechanisms may prevail (**Figure 1**). Indeed, a recent analysis based on artificial intelligence supports the notion that IVIG exerts pleiotropic effects, while distinct pathways, such as involving B cells or complement, were found to be more relevant for specific disorders (35). The inherent pluripotency of polyclonal IVIG and SCIG preparations might explain their efficacy in a magnitude of autoimmune and inflammatory diseases despite their heterogenous pathogenetic background. Furthermore, a potential mode of action might be related to the prominent plasmacytosis that is observed 7 days following IVIG infusion (36). This large “wave” of young mobilized plasmablasts could lead to a significant renewal of the repertoire of antibody-secreting cells through competition for bone marrow survival niches occupied by older plasmocytes. A “re-set” of the antibody repertoire in favour of non-pathogenic antibodies could thus partly explain the IVIG anti-inflammatory activity.

## IVIG and SCIG mechanisms - a source of inspiration

Insights on Fc-mediated MoAs of polyclonal immunoglobulin preparations stimulated the development of novel immunotherapeutic agents for the treatment of autoimmune and chronic inflammatory disorders. Together with co-workers, we recently showed that a recombinant IgG1 Fc hexamer (rFc-µTP-L309C) exhibits anti-inflammatory effects in the endogenous K/BxN mouse model of rheumatoid arthritis, which seems to involve the inhibition of neutrophil infiltration into the joints and the suppression of IL-1β production (37). A number of Fc-related biologics are currently tested in studies ranging from preclinical to ongoing phase 3 clinical trials and can be categorized into three classes: 1) recombinant Fc multimers, 2) neonatal Fc receptor (FcRn)-targeting therapeutics, and 3) Fc/Fcγ receptor (FcγR)-targeting therapeutics (reviewed in (38)).

Future immunotherapeutic drugs may also lean on the diversity of reported Fab-mediated and non-canonical MoAs of IVIG and SCIG preparations. For instance, IVIG has been shown to contain naturally-occurring functional antibodies against Siglecs (11, 12, 39), including Siglec-8 (11). Siglecs are single-pass cell surface receptors that regulate the activity of distinct subsets of immune cells (40–43), and induce cell death of cytokine-primed eosinophils (44) and neutrophils (45). Siglec receptors may thus provide a safeguard mechanism to limit undesired inflammation-associated tissue damage by activated granulocytes (46). AK002 (Lirentelimab), is a humanized non-fucosylated IgG1 antibody against Siglec-8 that is tested in several phase I and II studies for eosinophil- and mast cell-associated diseases (47). While AK002 may promote peripheral blood eosinophil depletion by antibody-dependent cell-mediated cytotoxicity (ADCC), direct death induction of activated eosinophils seems to be the main MoA in tissues, where CD16^+^ NK cells are scarce (47). Consistent with this notion, Siglec-8^+^ mast cells, which are inhibited but do not undergo apoptosis upon Siglec-8 signaling (42), were not depleted by AK002 in dissociated lung tissues (48).

The examples of Fc receptor-targeting biologics (38), and Lirentelimab (47), demonstrate the potential of deciphering IVIG or SCIG MoAs diversity, as a source of inspiration for the development of future immunotherapeutic drugs. However, while the antibody diversity of immunoglobulin preparations creates therapeutic value, the inherent complexity linked to their polyclonal composition comes not without challenges to scientists (15), and eventually also has clinical implications, at least for the treatment of select patient subsets (27, 32, 49). The repertoire of antibodies in IVIG or SCIG depends on the donor population (34), and differences between preparations and lots may exist in terms of the total repertoire (2), and eventually in respect to specific antibodies with relevant therapeutic functions. Indeed, in commercial immunoglobulin preparations variable titers of antibodies were reported not only in terms of specificities toward foreign antigens (49), but also toward autoantigens such as cytokines or immunoregulatory Fas or Siglec receptors (10, 49, 50). Furthermore, antagonistic or anti-idiotypic antibodies are found in IVIG (5, 9), and depending on their levels in select immunoglobulin preparations, these might potentially interfere with the action of specific antibodies and related MoAs (32). Future research is required to decipher the diversity of MoAs and to understand to what extent repertoire differences account for efficacy or resistance to IVIG or SCIG treatment. The identification of biomarkers and IVIG or SCIG lots with select antibody profiles might lead to better treatment responses and more personalized approaches to both antibody replacement (21), and anti-inflammatory immunotherapy.

## IgA, a curious case

In mammals, IgA is produced at higher levels than all other isotypes. IgA is routed *via* the polymeric Ig receptor (pIgR) at mucosal surfaces of the (small) intestine, in particular. The extensive glycosylation of IgA is thought to confer protease resistance, binding to bacterial adhesins, or serve as carbon source for microbiota (51). Indeed, recent research suggests that the role of secretory IgA (sIgA) is not restricted to immune defense, but extends to actively shaping the intestinal microbiome and its interactions with the host. Indeed, while IgA deficiency is thought to be partially compensated by increases of mucosal and serum levels of other antibody classes such as IgM or IgG (52), the underrepresentation in IgA deficient patients of some sIgA-coated species found in normal microbiota suggests a non-redundant role of sIgA in bacterial colonization (53).

sIgA has long been thought to protect from bacterial infection by immune exclusion relying on classical agglutination (54), influencing bacterial motility (55), or trapping bacteria in mucus (56). sIgA can also crosslink daughter cells of dividing bacteria resulting in ‘enchained growth’ (57), eventually leading to the formation of large bacterial clumps. While classical agglutination randomly associate bacteria of diverse origins and may be efficient at high bacterial densities, enchained growth will generate clumps with clonal structure and may be protective at lower bacterial densities (56). The mechanism of enchained growth by sIgA may further influence the composition of the microbiota in the gut by clonal extinction, growth rate selection (e.g. negative selective pressure related to pathogen-associated rapid growth) or horizontal gene transfer (56). In a recent study, it was shown that the overall intestinal IgA response to a single microbe involves multiple mechanisms in parallel, including altered gene transcription and protection from bile acids or bacteriophages, motility alterations and metabolic modulation (16).

## Secretory IgA: A taste for sweet

In the recent years, major progress in the characterization of the IgA repertoire was enabled by high-throughput methods, such as IgA-Seq and glycan array technologies (51, 58, 59). There is accumulating evidence that bacterial surface glycans are important targets of IgA (51). Commensal and pathogenic bacteria express a diversity of carbohydrates that are recognized by sIgA, as revealed by whole-microbiota enzymatic deglycosylation (60), as well as glycan array analysis of human (60) and mouse (61) sIgA. Indeed, the majority of surface-exposed bacterial antigens are glycans, which eventually evolved as products of a diversity-generating biosynthetic machinery (62). It has been postulated that glycobiology might become the ‘elephant in the room’ in sIgA biology (51).

Human intestinal IgA binds a wide subset of microbiota, including commensals from the four most frequent phyla (60). Given that humans have two IgA isotypes (in contrast to mice who only have a single IgA isotype), we recently compared IgA1 and IgA2 binding to gut bacteria (60). We noticed a predominance of IgA2^+^IgA1^+^ bacteria in the ileum, whereas IgA2 accounted for most colonic commensal binding (60). Interestingly, using glycan array technology we observed preferential binding of IgA2 compared to IgA1 to bacterial glycan structures, and this phenomenon was even more evident for Galα-structures, which are common in bacteria (60). Furthermore, a majority of IgM-bound bacteria was found to be recognized by both IgA1 and IgA2 (60). This observation is consistent with findings from Magri et al. who observed that bacteria bound to human secretory IgM (sIgM) are dually coated by IgA and are more diverse compared to IgA-only or uncoated bacteria (63). By this means, sIgM might support sIgA to anchor highly diverse commensals to mucus (63).

## A role for T cells in the generation of antibody responses to bacterial glycans

There is an ongoing discussion on the relative contributions of T-cell dependent and T-cell independent responses to IgA production in human and mice (51). It has been reported that a majority of microbiota species is coated by natural polyreactive sIgA derived from T cell-independent responses that bind in germline configuration, without the requirement for somatic mutations (61). However, in human adults, somatic mutations appear to contribute to the breath of commensals bound by intestinal IgA, and germline-like Ig conformations may rather present in young mice and very young children (51, 60, 64, 65). Although T cell-independent antibody responses to glycans have been reported many decades ago (66), more recent evidence suggests that T cells can be involved in carbohydrate-targeted antibody responses (67, 68), including to glycans in context of whole bacteria (57, 60, 69). Therefore, we share the opinion that it is *per se* not valid to define bacterial glycan-binding antibodies as predominantly T cell-independent (51). However, evidence suggests that both somatic hypermutation (70, 71), and class switch recombination to IgA (72, 73), can also occur in absence of T cells.

## A role for the microbiota in the generation of antibody responses to bacterial glycans

Pneumovax is a plain polysaccharide anti-pneumococcal vaccine inducing T-independent B cell responses. Recent data show that the B cell response against Pneumovax is dominated by large B cell clones from which monoclonal antibodies (mAbs) can be derived (74). Surprisingly, testing such mAbs against human microbiota revealed that they cross-react against a wide array of bacterial species from the gut flora (74). The data supports a model in which Pneumovax T-independent B cell responses most likely originate from prediversified mucosal immune responses against bacterial antigens that subsequently acquire pneumococcal cross-reactivity through somatic hypermutation. More work is necessary to determine whether this model applies to other conventional vaccines.

## A case for polyclonal IgA therapy

While the role played by IgA in the regulation of gut bacterial homeostasis appears now clearer, in our perspective there are currently no optimalcommercial IgA antibody preparations for oral use to test for their ability to regulate gut microbiome dysbiosis associated with primary antibody deficiencies. Despite IgA’s role in the growth and persistence of different species in the gut, only few studies have addressed the composition of the gut microbiome in the absence of IgA in PAD (53, 75–77). We and others reported a mild gut dysbiosis in patients with selective IgA deficiency (defined by lack of IgA in serum) (53, 76, 77). We observed that IgM only partially compensates for the lack of IgA because not all typical IgA targets are efficiently bound by IgM (53). In the gut microbiome of patients with CVID (characterized by low levels of IgG, IgA and/or IgM), we detected a reduction in bacterial species typically associated with health and enrichment in pro-inflammatory species (53). We and others recently observed that these alterations were correlated with elevated plasma levels of lipopolysaccharide (LPS) and sCD25, indicative of increased intestinal permeability and systemic immune activation (53, 78). Inability to produce functional Ig(s) is responsible for increased susceptibility to infections. Moreover, a proportion of PAD (both SIgAD and CVID) patients are at risk of developing non-infectious complications, including allergies, gastrointestinal inflammation, malignancies, and autoimmunity, causing significant morbidity and mortality (79).

Current systemic Ig replacement therapy in CVID restores only serum IgG levels eventually resulting in reduced numbers of respiratory infections. Yet, it still fails to protect against autoimmunity, allergy, gastrointestinal disorders and fatal liver disease (80). In addition, for SIgAD patients (normal IgG levels), this therapy is not an option. Thus, the only widely used remedies for gastrointestinal disorders are antibiotics (79), although they can magnify gut dysbiosis through the elimination of beneficial microbiota and the proliferation of drug-resistant bacteria (81, 82). Recent research showed that the SIgAD gut microbiome is, indeed, enriched in multidrug-resistant species (75). Treatment strategies that shield from PAD-related complications are, therefore, urgently needed. The protective role of oral IgA supplement in reducing bacterial translocation was reported in the neonatal rabbit model (83). While microbial translocation was thought to have a role in PAD immunopathology, approaches to correct gut dysbiosis and prevent translocation in PAD using IgA supplements remain to be assessed.

Clinical studies using oral administration of a plasma-derived and IgA-enriched IgG preparation, IgAbulin, has revealed promising results in children with chronic diarrhea (84), but mixed results in necrotizing enterocolitis (NEC), a life-threatening disorder primarily of preterm infants (27, 85). One limitation of plasma-derived IgA (pd-IgA) preparations is that they contain mainly IgA1 that is more susceptible to degradation by bacterial proteases (85). Furthermore, we observed that select bacteria such as Bacteroidetes and galactose-α terminated bacterial glycans are preferentially targeted by IgA2 (60). The better understanding of bacterial recognition by IgA1 and IgA2 will help to develop clinically more efficient oral IgA preparations for anti-infectious therapy.

Serum monomeric IgA exhibits anti-inflammatory properties (86–88), and IgA (89), or FcαRI targeting by monoclonal antibodies (90), has been shown to regulate the lifespan in particular of activated neutrophils. It remains to be shown if plasma-derived polyclonal IgA preparations might have therapeutic use for the treatment of inflammatory or autoimmune disorders.

## Enhancing the diversity of immunoglobulin preparations

Mild chemical modification of IVIG by agents such as ferrous ions, reactive oxygen species, heme or low pH-treatment leads to augmented immunoreactivity to peptide epitopes (91, 92), or shifted recognition patterns of glycans, as assessed by microarray technology (10). Not only may IVIG modification increase or enable *de novo* binding to bacterial antigens and intact bacteria (91), but also enhance capabilities to bind cytokines, complement components or danger molecules (93), or to regulate the life-span of leukocytes due to increased pro-apoptotic effects (10). Remarkably, modified IVIG showed superior therapeutic effects in several *in vivo* models of sepsis and aseptic systemic inflammatory response syndromes (93–95), or autoimmune diabetes (96), eventually due to the newly acquired antigen-binding and anti-inflammatory activities of modified immunoglobulin preparations. IgA reactivity can also be influenced by protein-modifying agents and exposure of colostrum and breast milk IgA to acidic pH was shown to expand the range of reactivities to bacterial antigens (from *Escherichia coli* and *Staphylococcus aureus*) and to viral antigens (from hepatitis C or D virus, human immunodeficiency virus type 2 and norovirus) (97). It remains to be shown to what extent intestinal pH differences or disturbances (e.g. hypochlorhydria) influence the local IgA repertoire and its effects on the host-microbial relationship.

Higher degree and non-site-specific antibody modification such as used for the generation of radioimmunoconjugates can be associated with reduced binding to the targeted antigen, eventually due to occupancy of the antigen-binding site (98). However, as revealed by crystallography, antibodies can adopt different binding-site conformations and thereby bind unrelated antigens, thus augmenting the effective size of the antibody repertoire (99). Mild chemical modification of antibodies is thought to increase conformational freedom and paratope flexibility by altering the nature of noncovalent forces responsible for antigen binding (91, 92). We recently proposed two additional effects of antibody modification: 1) release of pre-bound materials from body fluid-derived antibodies, thereby offering more options for specific interactions, and 2) partial denaturation leading to nonspecific interactions (100). Indeed, all these mechanisms combined are compatible with the observation that increased pro-apoptotic effects of modified IVIG involves enhanced reactivity to Fas receptor with concomitant loss of anti-Fas antibody neutralization capacities (10). Latter effect might involve the degradation of naturally-occurring blocking anti-Fas (9), or even of anti-idiotypic (5), antibodies in IVIG.

Mild chemical modification of antibodies seems to provide a mechanism for further diversification of antibody specificities, beyond somatic recombination, junctional diversity and somatic hypermutation (101). However, different protein-modifying conditions, such as ferrous ions, heme or low pH treatment, exhibit different effects on antibody reactivities (10, 97). Furthermore, additional immunoglobulin pre-treatments such as used for fractionation or virus-inactivation (102), might lead to different outcomes upon additional modification steps as shown for the induction of neutrophil death by different ferrous ion-exposed preparations (10). Future research, eventually involving reverse translational research approaches (103), will be required for the identification of chemical diversification strategies that enhance treatment benefits for select patient collectives amenable to antibody-based immunotherapy (27).

## Discussion

In the last decade, methodological advances have enabled greater insights into the human repertoire of antibodies, including sIgA. Research revealed that canonical and non-canonical effector functions of antibodies often act in concert to complete manifold tasks in immune defence, host-microbial symbiosis, or even immunoregulation. Insight into anti-inflammatory MoAs of IVIG has revealed the potential benefit of its pluripotency in targeting heterogenous disorders and served as a source for novel therapeutics. Nevertheless, research on IVIG mechanisms is often challenging due to the inherent complexity of polyclonal preparations (e.g. diversity, repertoires, blocking or anti-idiotypic antibodies). Limitations include the availability of suitable disease models (104, 105), and species differences of xenogeneic models to test human immunoglobulin products, which may affect treatment responses, such as of neutrophils (106). Furthermore, treatment responses to IVIG therapy can differ depending on patient characteristics and disease states (32), and the identification of suitable biomarkers and individual antibody “barcodes” (21, 26), may be needed for more personalized applications and immunoglobulin products. Despite these challenges, better understanding of the diversity of antibody repertoires holds promises for science, drug discovery and clinical application. Approaches to even enhance immunoglobulin diversity, such as by mild chemical modification, may provide novel avenues to antibody immunotherapy.

## Author contributions

All authors contributed to the article and approved the submitted version.

## References

[1] von Achenbach C Hevia Hernandez G von Gunten S. The choice between intravenous and subcutaneous immunoglobulins: Aspects for consideration. Pharmacology (2022) 107(11-12):556–63. doi: 10.1159/000527655 36349790

[2] Schneider C Smith DF Cummings RD Boligan KF Hamilton RG Bochner BS. The human IgG anti-carbohydrate repertoire exhibits a universal architecture and contains specificity for microbial attachment sites. Sci Transl Med (2015) 7:269ra1. doi: 10.1126/scitranslmed.3010524 PMC4864610 25568069

[3] von Gunten S Smith DF Cummings RD Riedel S Miescher S Schaub A. Intravenous immunoglobulin contains a broad repertoire of anticarbohydrate antibodies that is not restricted to the IgG2 subclass. J Allergy Clin Immunol (2009) 123(6):1268–76.e15. doi: 10.1016/j.jaci.2009.03.013 PMC2777748 19443021

[4] Lu LL Suscovich TJ Fortune SM Alter G. Beyond binding: Antibody effector functions in infectious diseases. Nat Rev Immunol (2018) 18:46–61. doi: 10.1038/NRI.2017.106 29063907 PMC6369690

[5] Schaub A von Gunten S Vogel M Wymann S Rüegsegger M Stadler BMM. Dimeric IVIG contains natural anti-Siglec-9 autoantibodies and their anti-idiotypes. Allergy (2011) 66:1030–7. doi: 10.1111/j.1398-9995.2011.02579.x 21385183

[6] Dimitrov JD Lacroix-Desmazes S. Noncanonical functions of antibodies. Trends Immunol (2020) 41:379–93. doi: 10.1016/J.IT.2020.03.006 32273170

[7] Mahendra A Sharma M Rao DN Peyron I Planchais C Dimitrov JD. Antibody-mediated catalysis: Induction and therapeutic relevance. Autoimmun Rev (2013) 12:648–52. doi: 10.1016/J.AUTREV.2012.10.009 23207286

[8] Prasad NK Papoff G Zeuner A Bonnin E Kazatchkine MD Ruberti G. Therapeutic preparations of normal polyspecific IgG (IVIg) induce apoptosis in human lymphocytes and monocytes: A novel mechanism of action of IVIg involving the fas apoptotic pathway. J Immunol (1998) 161:3781–90. 9759905

[9] Altznauer F von Gunten S Späth P Simon H-U. Concurrent presence of agonistic and antagonistic anti-CD95 autoantibodies in intravenous ig preparations. J Allergy Clin Immunol (2003) 112:1185–90. doi: 10.1016/j.jaci.2003.09.045 14657880

[10] Graeter S Schneider C Verschoor D von Däniken S Seibold F Yawalkar N. Enhanced pro-apoptotic effects of Fe(II)-modified IVIG on human neutrophils. Front Immunol (2020) 11:973. doi: 10.3389/fimmu.2020.00973 32508840 PMC7248553

[11] von Gunten S Vogel M Schaub A Stadler BM Miescher S Crocker PR. Intravenous immunoglobulin preparations contain anti-Siglec-8 autoantibodies. J Allergy Clin Immunol (2007) 119:1005–11. doi: 10.1016/j.jaci.2007.01.023 17337295

[12] von Gunten S Schaub A Vogel M Stadler BMBM Miescher S Simon H-U. Immunologic and functional evidence for anti-Siglec-9 autoantibodies in intravenous immunoglobulin preparations. Blood (2006) 108:4255–9. doi: 10.1182/blood-2006-05-021568 16902148

[13] Graeter S Simon H-U von Gunten S. Granulocyte death mediated by specific antibodies in intravenous immunoglobulin (IVIG). Pharmacol Res (2020) 154:104168. doi: 10.1016/j.phrs.2019.02.007 30738127

[14] Cousens LP Tassone R Mazer BD Ramachandiran V Scott DW de Groot AS. Tregitope update: Mechanism of action parallels IVIg. Autoimmun Rev (2013) 12:436–43. doi: 10.1016/J.AUTREV.2012.08.017 22944299

[15] von Gunten S Shoenfeld Y Blank M Branch DR Vassilev T Käsermann F. IVIG pluripotency and the concept of fc-sialylation: challenges to the scientist. Nat Rev Immunol (2014) 14:349. doi: 10.1038/nri3401-c1 24762829

[16] Rollenske T Burkhalter S Muerner L von Gunten S Lukasiewicz J Wardemann H. Parallelism of intestinal secretory IgA shapes functional microbial fitness. Nature (2021) 598:657–61. doi: 10.1038/s41586-021-03973-7 34646015

[17] von Gunten S Cortinas-Elizondo F Kollarik M Beisswenger C Lepper PMM von Gunten S. Mechanisms and potential therapeutic targets in allergic inflammation: Recent insights. Allergy (2013) 68:1487–98. doi: 10.1111/all.12312 24215555

[18] Cohen IR Efroni S. The immune system computes the state of the body: Crowd wisdom, machine learning, and immune cell reference repertoires help manage inflammation. Front Immunol (2019) 10:10. doi: 10.3389/FIMMU.2019.00010 30723470 PMC6349705

[19] Cohen IR. Activation of benign autoimmunity as both tumor and autoimmune disease immunotherapy: A comprehensive review. J Autoimmun (2014) 54:112–7. doi: 10.1016/J.JAUT.2014.05.002 24924121

[20] Cohen IR. Real and artificial immune systems: Computing the state of the body. Nat Rev Immunol (2007) 7:569–74. doi: 10.1038/nri2102 17558422

[21] Jandus P Frias Boligan K Smith DF de Graauw E Grimbacher B Jandus C. The architecture of the IgG anti-carbohydrate repertoire in primary antibody deficiencies (PADs). Blood (2019) 134:1941–50. doi: 10.1182/blood.2019001705 PMC6887115 31537530

[22] Mereiter S Polom K Williams C Polonia A Guergova-Kuras M Karlsson NG. The thomsen-friedenreich antigen: A highly sensitive and specific predictor of microsatellite instability in gastric cancer. J Clin Med (2018) 7(9):256. doi: 10.3390/JCM7090256 30189652 PMC6162870

[23] Santos-Silva F Fonseca A Caffrey T Carvalho F Mesquita P Reis C. Thomsen-friedenreich antigen expression in gastric carcinomas is associated with MUC1 mucin VNTR polymorphism. Glycobiology (2005) 15:511–7. doi: 10.1093/GLYCOB/CWI027 15604091

[24] zum Büschenfelde CM Feuerstacke Y Götze KS Scholze K Peschel C. GM1 expression of non-hodgkin’s lymphoma determines susceptibility to rituximab treatment. Cancer Res (2008) 68:5414–22. doi: 10.1158/0008-5472.CAN-07-5601 18593944

[25] Mortaz E Tabarsi P Mansouri D Khosravi A Garssen J Velayati A. Cancers related to immunodeficiencies: Update and perspectives. Front Immunol (2016) 7:365. doi: 10.3389/FIMMU.2016.00365 27703456 PMC5028721

[26] Luetscher RND McKitrick TR Gao C Mehta AY McQuillan AM Kardish R. Unique repertoire of anti-carbohydrate antibodies in individual human serum. Sci Rep (2020) 10:15436. doi: 10.1038/s41598-020-71967-y 32963315 PMC7509809

[27] Späth PJ Schneider C von Gunten S. Clinical use and therapeutic potential of IVIG/SCIG, plasma-derived IgA or IgM, and other alternative immunoglobulin preparations. Arch Immunol Ther Exp (2017) 65:215–31. doi: 10.1007/s00005-016-0422-x 27638480

[28] Fadlallah J Sterlin D Fieschi C Parizot C Dorgham K el Kafsi H. Synergistic convergence of microbiota-specific systemic IgG and secretory IgA. J Allergy Clin Immunol (2019) 143:1575–1585.e4. doi: 10.1016/J.JACI.2018.09.036 30554723

[29] Kazatchkine MD Kaveri SV. Immunomodulation of autoimmune and inflammatory diseases with intravenous immune globulin. N Engl J Med (2001) 345:747–55. doi: 10.1056/NEJMRA993360 11547745

[30] Galeotti C Kaveri SV Bayry J. IVIG-mediated effector functions in autoimmune and inflammatory diseases. Int Immunol (2017) 29:491–8. doi: 10.1093/INTIMM/DXX039 28666326

[31] Ballow M. Mechanisms of immune regulation by IVIG. Curr Opin Allergy Clin Immunol (2014) 14:509–15. doi: 10.1097/ACI.0000000000000116 25337683

[32] von Gunten S Simon H-U. Cell death modulation by intravenous immunoglobulin. J Clin Immunol (2010) 30(Suppl 1):S24–30. doi: 10.1007/s10875-010-9411-8 20405180

[33] Séïté JF Goutsmedt C Youinou P Pers JO Hillion S. Intravenous immunoglobulin induces a functional silencing program similar to anergy in human b cells. J Allergy Clin Immunol (2014) 133(1):181–8.e1–9. doi: 10.1016/J.JACI.2013.08.042 24139609

[34] Negi VS Elluru S Sibéril S Graff-Dubois S Mouthon L Kazatchkine MD. Intravenous immunoglobulin: An update on the clinical use and mechanisms of action. J Clin Immunol (2007) 27:233–45. doi: 10.1007/S10875-007-9088-9 17351760

[35] Segú-Vergés C Caño S Calderón-Gómez E Bartra H Sardon T Kaveri S. Systems biology and artificial intelligence analysis highlights the pleiotropic effect of IVIg therapy in autoimmune diseases with a predominant role on b cells and complement system. Front Immunol (2022) 13:901872. doi: 10.3389/FIMMU.2022.901872 36248801 PMC9563374

[36] Mori I Parizot C Dorgham K Demeret S Amoura Z Bolgert F. Prominent plasmacytosis following intravenous immunoglobulin correlates with clinical improvement in Guillain-Barré syndrome. PloS One (2008) 3(5):e2109. doi: 10.1371/JOURNAL.PONE.0002109 18461177 PMC2362102

[37] Almizraq RJ Frias Boligan K Lewis BJB Cen S Whetstone H Spirig R. Modulation of neutrophil function by recombinant human IgG1 fc hexamer in the endogenous K/BxN mouse model of rheumatoid arthritis. Pharmacology (2023) 108(2):176–87. doi: 10.1159/000528780 PMC10015763 36696888

[38] Zuercher AW Spirig R Baz Morelli A Rowe T Käsermann F. Next-generation fc receptor-targeting biologics for autoimmune diseases. Autoimmun Rev (2019) 18(10):102366. doi: 10.1016/J.AUTREV.2019.102366 31404703

[39] von Gunten S Simon H-U. Granulocyte death regulation by naturally occurring autoantibodies. Adv Exp Med Biol (2012) 750:157–72. doi: 10.1007/978-1-4614-3461-0_12 22903673

[40] Haas Q Markov N Muerner L Rubino V Benjak A Haubitz M. Siglec-7 represents a glyco-immune checkpoint on for non-exhausted effector memory CD8+ T cells with high functional and metabolic capacities. Front Immunol (2022) 13:996746. doi: 10.3389/fimmu.2022.996746 36211376 PMC9540514

[41] Duan S Paulson JC. Siglecs as immune cell checkpoints in disease. Annu Rev Immunol (2020) 38:365–95. doi: 10.1146/ANNUREV-IMMUNOL-102419-035900 31986070

[42] Yokoi H Choi OH Hubbard W Lee H-S Canning BJBJ Lee HH. Inhibition of FcepsilonRI-dependent mediator release and calcium flux from human mast cells by sialic acid-binding immunoglobulin-like lectin 8 engagement. J Allergy Clin Immunol (2008) 121(2):499–505.e1. doi: 10.1016/j.jaci.2007.10.004 18036650

[43] Haas Q Boligan KF Jandus C Schneider C Simillion C Stanczak MA. Siglec-9 regulates an effector memory CD8+ T-cell subset that congregates in the melanoma tumor microenvironment. Cancer Immunol Res (2019) 7:707–18. doi: 10.1158/2326-6066.CIR-18-0505 30988027

[44] Nutku E Aizawa H Hudson SA Bochner BS. Ligation of siglec-8: A selective mechanism for induction of human eosinophil apoptosis. Blood (2003) 101:5014–20. doi: 10.1182/BLOOD-2002-10-3058 12609831

[45] von Gunten S Yousefi S Seitz M Jakob SM Schaffner T Seger R. Siglec-9 transduces apoptotic and nonapoptotic death signals into neutrophils depending on the proinflammatory cytokine environment. Blood (2005) 106:1423–31. doi: 10.1182/blood-2004-10-4112 15827126

[46] Kaufmann T Simon H-U. Pharmacological induction of granulocyte cell death as therapeutic strategy. Annu Rev Pharmacol Toxicol (2022) 63:231–47. doi: 10.1146/ANNUREV-PHARMTOX-051921-115130 36028226

[47] Youngblood BA Leung J Falahati R Williams J Schanin J Brock EC. Discovery, function, and therapeutic targeting of siglec-8. Cells (2020) 10:1–14. doi: 10.3390/CELLS10010019 33374255 PMC7823959

[48] Kerr SC Gonzalez JR Schanin J Peters MC Lambrecht BN Brock EC. An anti-siglec-8 antibody depletes sputum eosinophils from asthmatic subjects and inhibits lung mast cells. Clin Exp Allergy (2020) 50:904–14. doi: 10.1111/CEA.13681 PMC7610812 32542913

[49] Simon HU Späth PJ. IVIG–mechanisms of action. Allergy (2003) 58:543–52. doi: 10.1034/J.1398-9995.2003.00239.X 12823109

[50] Reipert BM Stellamor MT Poell M Ilas J Sasgary M Reipert S. Variation of anti-fas antibodies in different lots of intravenous immunoglobulin. Vox Sang (2008) 94:334–41. doi: 10.1111/J.1423-0410.2008.001036.X 18266779

[51] Pabst O Slack E. IgA and the intestinal microbiota: The importance of being specific. Mucosal Immunol (2020) 13:12–21. doi: 10.1038/S41385-019-0227-4 31740744 PMC6914667

[52] Harriman GR Bogue M Rogers P Finegold M Pacheco S Bradley A. Targeted deletion of the IgA constant region in mice leads to IgA deficiency with alterations in expression of other ig isotypes. J Immunol (1999) 162(5):2521–9. 10072491

[53] Fadlallah J el Kafsi H Sterlin D Juste C Parizot C Dorgham K. Microbial ecology perturbation in human IgA deficiency. Sci Transl Med (2018) 10(439):eaan1217. doi: 10.1126/SCITRANSLMED.AAN1217 29720448

[54] Mantis NJ Rol N Corthésy B. Secretory IgA’s complex roles in immunity and mucosal homeostasis in the gut. Mucosal Immunol (2011) 4:603–11. doi: 10.1038/MI.2011.41 PMC3774538 21975936

[55] Forbes SJ Eschmann M Mantis NJ. Inhibition of salmonella enterica serovar typhimurium motility and entry into epithelial cells by a protective antilipopolysaccharide monoclonal immunoglobulin a antibody. Infect Immun (2008) 76:4137–44. doi: 10.1128/IAI.00416-08 PMC2519396 18625740

[56] Hoces D Arnoldini M Diard M Loverdo C Slack E. Growing, evolving and sticking in a flowing environment: Understanding IgA interactions with bacteria in the gut. Immunology (2020) 159:52–62. doi: 10.1111/IMM.13156 31777063 PMC6904610

[57] Moor K Diard M Sellin ME Felmy B Wotzka SY Toska A. High-avidity IgA protects the intestine by enchaining growing bacteria. Nature (2017) 544:498–502. doi: 10.1038/NATURE22058 28405025

[58] Macpherson AJ Yilmaz B Limenitakis JP Ganal-Vonarburg SC. IgA function in relation to the intestinal microbiota. Annu Rev Immunol (2018) 36:359–81. doi: 10.1146/ANNUREV-IMMUNOL-042617-053238 29400985

[59] Pashova S Schneider C von Gunten S Pashov A. Antibody repertoire profiling with mimotope arrays. Hum Vaccin Immunother (2017) 13:314–22. doi: 10.1080/21645515.2017 PMC5328411 27929733

[60] Sterlin D Fadlallah J Adams O Fieschi C Parizot C Dorgham K. Human IgA binds a diverse array of commensal bacteria. J Exp Med (2020) 217:e20181635. doi: 10.1084/jem.20181635 31891367 PMC7062531

[61] Bunker JJ Erickson SA Flynn TM Henry C Koval JC Meisel M. Natural polyreactive IgA antibodies coat the intestinal microbiota. Science (2017) 358(6361):eaan6619. doi: 10.1126/SCIENCE.AAN6619 28971969 PMC5790183

[62] Mostowy RJ Holt KE. Diversity-generating machines: Genetics of bacterial sugar-coating. Trends Microbiol (2018) 26:1008–21. doi: 10.1016/J.TIM.2018.06.006 PMC6249986 30037568

[63] Magri G Comerma L Pybus M Sintes J Lligé D Segura-Garzón D. Human secretory IgM emerges from plasma cells clonally related to gut memory b cells and targets highly diverse commensals. Immunity (2017) 47:118–134.e8. doi: 10.1016/J.IMMUNI.2017.06.013 28709802 PMC5519504

[64] Lindner C Wahl B Föhse L Suerbaum S Macpherson AJ Prinz I. Age, microbiota, and T cells shape diverse individual IgA repertoires in the intestine. J Exp Med (2012) 209:365–77. doi: 10.1084/JEM.20111980 PMC3280880 22249449

[65] Lindner C Thomsen I Wahl B Ugur M Sethi MK Friedrichsen M. Diversification of memory b cells drives the continuous adaptation of secretory antibodies to gut microbiota. Nat Immunol (2015) 16:880–8. doi: 10.1038/NI.3213 26147688

[66] Hiernaux JR Jones JM Rudbach JA Rollwagen F Baker PJ. Antibody response of immunodeficient (xid) CBA/N mice to escherichia coli 0113 lipopolysaccharide, a thymus-independent antigen. J Exp Med (1983) 157:1197–207. doi: 10.1084/JEM.157.4.1197 PMC2186980 6187886

[67] Avci FY Li X Tsuji M Kasper DL. A mechanism for glycoconjugate vaccine activation of the adaptive immune system and its implications for vaccine design. Nat Med (2011) 17:1602–9. doi: 10.1038/nm.2535 PMC3482454 22101769

[68] Li S Rouphael N Duraisingham S Romero-Steiner S Presnell S Davis C. Molecular signatures of antibody responses derived from a systems biology study of five human vaccines. Nat Immunol (2014) 15:195–204. doi: 10.1038/ni.2789 24336226 PMC3946932

[69] Moor K Wotzka SY Toska A Diard M Hapfelmeier S Slack E. Peracetic acid treatment generates potent inactivated oral vaccines from a broad range of culturable bacterial species. Front Immunol (2016) 7:34. doi: 10.3389/FIMMU.2016.00034 26904024 PMC4749699

[70] Weller S Faili A Garcia C Braun MC le Deist F de Saint Basile G. CD40-CD40L independent ig gene hypermutation suggests a second b cell diversification pathway in humans. Proc Natl Acad Sci USA (2001) 98:1166–70. doi: 10.1073/PNAS.98.3.1166 PMC14726 11158612

[71] Scheeren FA Nagasawa M Weijer K Cupedo T Kirberg J Legrand N. T Cell-independent development and induction of somatic hypermutation in human IgM+ IgD+ CD27+ b cells. J Exp Med (2008) 205:2033–42. doi: 10.1084/JEM.20070447 PMC2526198 18695003

[72] Cerutti A. The regulation of IgA class switching. Nat Rev Immunol (2008) 8:421–34. doi: 10.1038/NRI2322 PMC3062538 18483500

[73] Lycke NY Bemark M. The regulation of gut mucosal IgA b-cell responses: Recent developments. Mucosal Immunol (2017) 10:1361–74. doi: 10.1038/MI.2017.62 28745325

[74] Weller S Sterlin D Fadeev T Coignard E Verge de los Aires A Goetz C. T-Independent responses to polysaccharides in humans mobilize marginal zone b cells prediversified against gut bacterial antigens. Sci Immunol (2023) 8(79):eade1413. doi: 10.1126/SCIIMMUNOL.ADE1413 36706172 PMC7614366

[75] Moll JM Myers PN Zhang C Eriksen C Wolf J Appelberg KS. Gut microbiota perturbation in IgA deficiency is influenced by IgA-autoantibody status. Gastroenterology (2021) 160:2423–2434.e5. doi: 10.1053/J.GASTRO.2021.02.053 33662387

[76] Jørgensen SF Holm K Macpherson ME Storm-Larsen C Kummen M Fevang B. Selective IgA deficiency in humans is associated with reduced gut microbial diversity. J Allergy Clin Immunol (2019) 143:1969–1971.e11. doi: 10.1016/J.JACI.2019.01.019 30707969

[77] Catanzaro JR Strauss JD Bielecka A Porto AF Lobo FM Urban A. IgA-deficient humans exhibit gut microbiota dysbiosis despite secretion of compensatory IgM. Sci Rep (2019) 9(1):13574. doi: 10.1038/S41598-019-49923-2 31537840 PMC6753154

[78] Jørgensen SF Trøseid M Kummen M Anmarkrud JA Michelsen AE Osnes LT. Altered gut microbiota profile in common variable immunodeficiency associates with levels of lipopolysaccharide and markers of systemic immune activation. Mucosal Immunol (2016) 9:1455–65. doi: 10.1038/MI.2016.18 26982597

[79] Demirdag YY Gupta S. Update on infections in primary antibody deficiencies. Front Immunol (2021) 12:634181. doi: 10.3389/FIMMU.2021.634181 33643318 PMC7905085

[80] Resnick ES Moshier EL Godbold JH Cunningham-Rundles C. Morbidity and mortality in common variable immune deficiency over 4 decades. Blood (2012) 119:1650–7. doi: 10.1182/BLOOD-2011-09-377945 PMC3286343 22180439

[81] Kang K Imamovic L Misiakou MA Bornakke Sørensen M Heshiki Y Ni Y. Expansion and persistence of antibiotic-specific resistance genes following antibiotic treatment. Gut Microbes (2021) 13:1–19. doi: 10.1080/19490976.2021.1900995 PMC8018486 33779498

[82] van der Helm E Imamovic L Ellabaan MMH van Schaik W Koza A Sommer MOA. Rapid resistome mapping using nanopore sequencing. Nucleic Acids Res (2017) 45(8):e61. doi: 10.1093/NAR/GKW1328 28062856 PMC5416750

[83] Maxson RT Jackson RJ Smith SD. The protective role of enteral IgA supplementation in neonatal gut origin sepsis. J Pediatr Surg (1995) 30:231–4. doi: 10.1016/0022-3468(95)90566-9 7738744

[84] Casswall TH Hammarström L Veress B Nord CE Bogstedt A Brockstedt U. Oral IgA-IgG treatment of chronic non-specific diarrhoea in infants and children. Acta Paediatr (1996) 85:1126–8. doi: 10.1111/J.1651-2227.1996.TB14231.X 8888931

[85] Langereis JD van der Flier M de Jonge MI. Limited innovations after more than 65 years of immunoglobulin replacement therapy: Potential of IgA- and IgM-enriched formulations to prevent bacterial respiratory tract infections. Front Immunol (2018) 9:1925. doi: 10.3389/FIMMU.2018.01925 30190722 PMC6115500

[86] Mkaddem Sb Christou I Rossato E Berthelot L Lehuen AA Monteiro RC. IgA, IgA receptors, and their anti-inflammatory properties. Curr Top Microbiol Immunol (2014) 382:221–35. doi: 10.1007/978-3-319-07911-0_10 25116102

[87] Jacob CMA Pastorino AC Fahl K Carneiro-Sampaio M Monteiro RC. Autoimmunity in IgA deficiency: Revisiting the role of IgA as a silent housekeeper. J Clin Immunol (2008) Suppl 1:S56–61. doi: 10.1007/s10875-007-9163-2 18202833

[88] Monteiro RC. Role of IgA and IgA fc receptors in inflammation. J Clin Immunol (2010) 30:1–9. doi: 10.1007/s10875-009-9338-0 19834792

[89] Wehrli M Schneider C Cortinas-Elizondo F Verschoor D Frias Boligan K Adams OJ. IgA triggers cell death of neutrophils when primed by inflammatory mediators. J Immunol (2020) 205:2640–8. doi: 10.4049/JIMMUNOL.1900883 33008951

[90] Wehrli M Cortinas-Elizondo F Hlushchuk R Daudel F Villiger PMM Miescher S. Human IgA fc receptor FcalphaRI (CD89) triggers different forms of neutrophil death depending on the inflammatory microenvironment. J Immunol (2014) 193:5649–59. doi: 10.4049/jimmunol.1400028 25339672

[91] Dimitrov JD Roumenina LT Doltchinkova VR Mihaylova NM Lacroix-Desmazes S Kaveri SV. Antibodies use heme as a cofactor to extend their pathogen elimination activity and to acquire new effector functions. J Biol Chem (2007) 282:26696–706. doi: 10.1074/JBC.M702751200 17636257

[92] Dimitrov JD Ivanovska ND Lacroix-Desmazes S Doltchinkova VR Kaveri SV Vassilev TL. Ferrous ions and reactive oxygen species increase antigen-binding and anti-inflammatory activities of immunoglobulin G. J Biol Chem (2006) 281:439–46. doi: 10.1074/JBC.M509190200 16246843

[93] Djoumerska-Alexieva I Roumenina L Pashov A Dimitrov J Hadzhieva M Lindig S. Intravenous immunoglobulin with enhanced polyspecificity improves survival in experimental sepsis and aseptic systemic inflammatory response syndromes. Mol Med (2015) 21:2–10. doi: 10.2119/molmed.2014.00224 PMC4982479 26701312

[94] Djoumerska-Alexieva I Roumenina LT Stefanova T Vassilev T Dimitrov JD. Heme-exposed pooled therapeutic IgG improves endotoxemia survival. Inflammation (2017) 40:117–22. doi: 10.1007/S10753-016-0460-X 27796617

[95] Djoumerska-Alexieva IK Dimitrov JD Nacheva J Kaveri SV Vassilev TL. Protein destabilizing agents induce polyreactivity and enhanced immunomodulatory activity in IVIg preparations. Autoimmunity (2009) 42:365–7. doi: 10.1080/08916930902832181 19811303

[96] Pavlovic S Zdravkovic N Dimitrov JD Djukic A Arsenijevic N Vassilev TL. Intravenous immunoglobulins exposed to heme (heme IVIG) are more efficient than IVIG in attenuating autoimmune diabetes. Clin Immunol (2011) 138:162–71. doi: 10.1016/J.CLIM.2010.10.010 21123117

[97] Gorshkova EN Pashova S Vasilenko EA Tchurina TS Razzorenova EA Starkina OV. Induced polyspecificity of human secretory immunoglobulin a antibodies: Is it possible to improve their ability to bind pathogens? Pharmacology (2021) 107(7-8):341–50. doi: 10.1159/000520343 34864734

[98] Basaco T Pektor S Bermudez JM Meneses N Heller M Galván JA. Evaluation of radiolabeled girentuximab *In vitro* and *In vivo* . Pharm (Basel) (2018) 11(4):132. doi: 10.3390/PH11040132 PMC6316122 30487460

[99] James LC Roversi P Tawfik DS. Antibody multispecificity mediated by conformational diversity. Science (2003) 299:1362–7. doi: 10.1126/SCIENCE.1079731 12610298

[100] Gorochov G von Gunten S. Diversification of IgA antibody specificities by mild chemical modification? Pharmacology (2022) 107:339–40. doi: 10.1159/000524041 35358972

[101] Kanyavuz A Marey-Jarossay A Lacroix-Desmazes S Dimitrov JD. Breaking the law: Unconventional strategies for antibody diversification. Nat Rev Immunol (2019) 19:355–68. doi: 10.1038/S41577-019-0126-7 30718829

[102] Djoumerska I Tchorbanov A Pashov A Vassilev T. The autoreactivity of therapeutic intravenous immunoglobulin (IVIG) preparations depends on the fractionation methods used. Scand J Immunol (2005) 61:357–63. doi: 10.1111/J.1365-3083.2005.01568.X 15853919

[103] von Gunten S. The future of pharmacology: Towards more personalized pharmacotherapy and reverse translational research. Pharmacology (2020) 105:1–2. doi: 10.1159/000505216 31822007

[104] Quast I Keller CW Weber P Schneider C von Gunten S Lünemann JD. Protection from experimental autoimmune encephalomyelitis by polyclonal IgG requires adjuvant-induced inflammation. J Neuroinflamm (2016) 13:42. doi: 10.1186/s12974-016-0506-x PMC4758141 26893156

[105] von Gunten S Wehrli M Simon H-U. Cell death in immune thrombocytopenia: Novel insights and perspectives. Semin Hematol (2013) 50(Suppl 1):S109–15. doi: 10.1053/j.seminhematol.2013.03.016 23664507

[106] Schneider C Wicki S Graeter S Timcheva TM Keller CW Quast I. IVIG regulates the survival of human but not mouse neutrophils. Sci Rep (2017) 7:1296. doi: 10.1038/s41598-017-01404-0 28465620 PMC5430961

